# The Good the Bad and the Ugly of Glycosaminoglycans in Tissue Engineering Applications

**DOI:** 10.3390/ph10020054

**Published:** 2017-06-13

**Authors:** Bethanie I. Ayerst, Catherine L.R. Merry, Anthony J. Day

**Affiliations:** 1 Wellcome Trust Centre for Cell-Matrix Research, Division of Cell-Matrix Biology & Regenerative Medicine, School of Biology, Faculty of Biology, Medicine & Health, The University of Manchester, Manchester Academic Health Science Centre, Manchester M13 9PL, UK; bethayerst@me.com; 2 Stem Cell Glycobiology Group, Wolfson Centre for Stem Cells, Tissue Engineering & Modelling (STEM), Centre for Biomolecular Sciences, University of Nottingham, University Park, Nottingham NG7 2RD, UK; Cathy.Merry@nottingham.ac.uk

**Keywords:** glycosaminoglycans, heparin, heparan sulfate, cartilage, mesenchymal stem cells, tissue engineering, growth factors, growth differentiation factor 5, GDF5

## Abstract

High sulfation, low cost, and the status of heparin as an already FDA- and EMA- approved product, mean that its inclusion in tissue engineering (TE) strategies is becoming increasingly popular. However, the use of heparin may represent a naïve approach. This is because tissue formation is a highly orchestrated process, involving the temporal expression of numerous growth factors and complex signaling networks. While heparin may enhance the retention and activity of certain growth factors under particular conditions, its binding ‘promiscuity’ means that it may also inhibit other factors that, for example, play an important role in tissue maintenance and repair. Within this review we focus on articular cartilage, highlighting the complexities and highly regulated processes that are involved in its formation, and the challenges that exist in trying to effectively engineer this tissue. Here we discuss the opportunities that glycosaminoglycans (GAGs) may provide in advancing this important area of regenerative medicine, placing emphasis on the need to move away from the common use of heparin, and instead focus research towards the utility of specific GAG preparations that are able to modulate the activity of growth factors in a more controlled and defined manner, with less off-target effects.

## 1. Introduction

Tissue Engineering (TE) is a multidisciplinary research area involving combinations of biomaterials, differentiating cells, and bioactive factors to produce functional tissues and organs. Articular cartilage has limited ability to regenerate, and has therefore become a key target for TE strategies. However, despite sustained efforts, most techniques to date have failed to generate a truly biomimetic articular cartilage. Here we review the biology behind this highly structured tissue, the challenges that have been faced in trying to engineer cartilage, and the advancements that are currently being made within this field. In particular, we highlight the exciting potential that incorporating GAGs into cartilage TE strategies may provide, as well as the limitations and drawbacks that they may introduce if not used in a controlled manner.

## 2. Articular Cartilage

**Figure 1:**
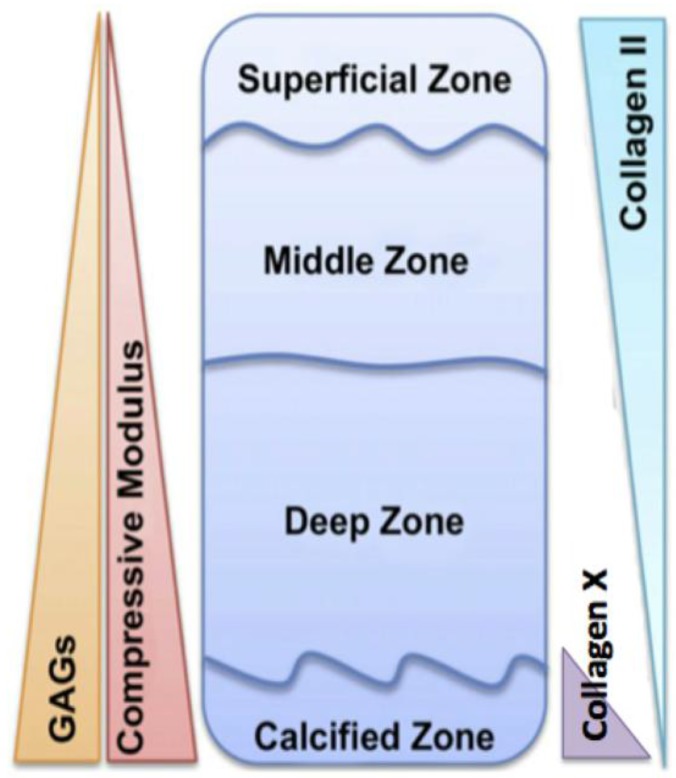
Schematic demonstrating the layered structure of articular cartilage. The transition from the superficial to the calcified zone is characterised by an increase in GAG content and compressive strength, but a decrease in collagen II. Collagen X is usually only found in the calcified zone of healthy articular cartilage. Figure adapted from [8,11]. Figure not to scale.

Articular (hyaline) cartilage is a predominantly alymphatic, aneural, and avascular tissue, consisting of chondrocytes, which make up only 1–5% of its total volume, embedded within an extensive extracellular matrix (ECM) (as reviewed [1,2,3]). Being widely dispersed, chondrocytes are responsible for the synthesis, maintenance and turnover of the ECM, in response to signals from growth factors, cytokines, adipokines, inflammatory mediators and matrix fragments (see [4]). Type II collagen and the proteoglycan (PG) aggrecan, play the most important structural roles in the ECM of cartilage, together forming a hydrodynamic, tensile meshwork, with a high compressive strength [2,5]. Given these properties, the tissue plays a vital role in load-bearing joints, and provides an almost frictionless surface to articulating bones [6]. The ECM of articular cartilage is also highly structured and organised, and can be divided into four spatially distinct regions, namely the superficial, middle, deep, and calcified zones (see [6,7,8]). As shown in Figure 1, each zone is characterised by unique ECM compositions, mechanical properties and cellular organisation. Studies have demonstrated that even small changes in the ECM of articular cartilage can lead to disruption in its mechanical properties, highlighting that structural organisation and continual remodelling of ECM molecules by chondrocytes is crucial to the proper functioning of the tissue [9,10].

### 2.1. Formation

**Figure 2:**
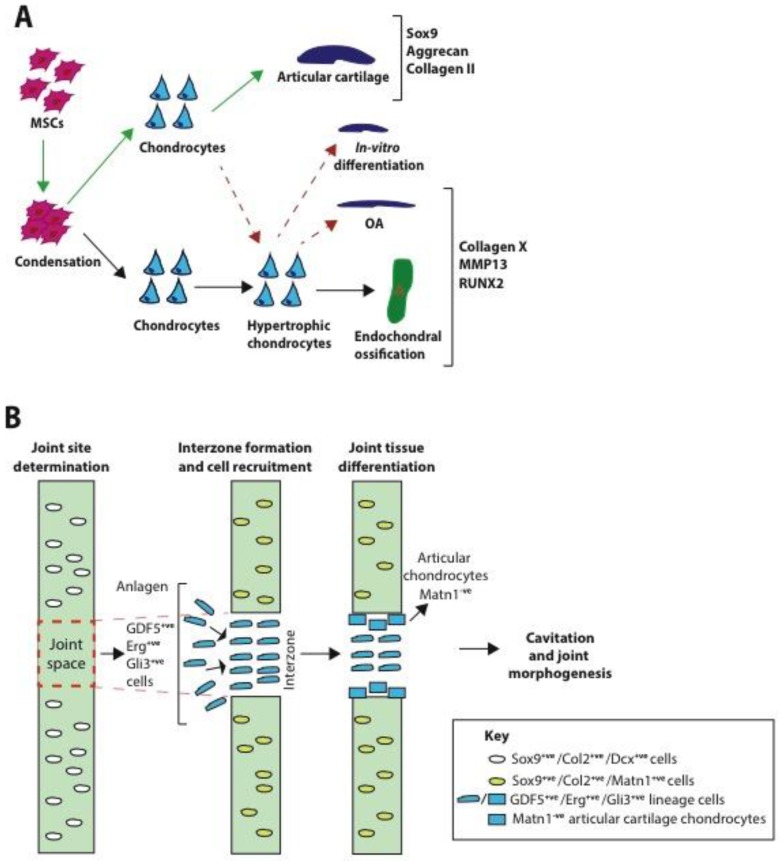
Articular cartilage formation. (**A**) Chondrogenesis is initiated during limb bud development with the condensation of MSCs. These progenitors then differentiate into chondrocytes that go on to form permanent articular cartilage, or into chondrocytes that eventually undergo hypertrophy and endochondral ossification. The complex spatiotemporal cues required to maintain chondrocytes in a permanent articular cartilage-like phenotype are not yet fully understood, and as such, the majority of regeneration strategies currently result in the formation of hypertrophic rather than hyaline-like tissue; (**B**) Articular cartilage is thought to originate from a distinct population of MSCs during limb joint formation; GDF5/Erg/Gli3 expressing cells within the joint space define the initial interzone MSC population, and this population becomes sandwiched between the two cartilage anlagen, while anlagen bound chondrocytes turn on expression of Matn1. GDF5/Erg/Gli3 expressing cells adjacent to their respective cartilaginous anlagen, but which have never expressed Matn1, then go on to differentiate into articular chondrocytes. (**A**) adapted from [20]; (**B**) adapted from [12]. Abbreviations: Dcx, doublecortin; OA, osteoarthritis.

In many respects, our understanding of the mechanisms involved in the formation, organisation and maintenance of articular cartilage remain unclear and challenging [12]. Much of the progress that has been made to date has come from research into the formation of the axial and appendicular skeletons, which is initiated by limb bud development, through the highly regulated process of endochondral ossification [13,14,15,16,17,18,19]. This process begins early during embryogenesis with the aggregation and condensation of mesenchymal stem/stromal cells (MSCs) derived from the mesoderm germ layer. MSCs within these condensations then proliferate, increasing the cell density within the condensate, before undergoing differentiation into prechondrogenic cells that form a cartilage template/anlagen. At this stage the prechondrogenic cells are then thought to either further differentiate into chondrocytes that produce large amounts of ECM and form permanent hyaline cartilage, or into proliferating chondrocytes that form the growth plate and eventually undergo hypertrophy [19] (Figure 2A).

Accumulating evidence suggests that permanent hyaline cartilage originates from a distinct population of MSCs, referred to as interzone cells (see [12,21]). These cells are found sandwiched between cartilage anlagen and are characterised by expression of genes such as erythroblast transformation-specific-related gene (Erg), growth differentiation factor 5 (GDF5), and GLI family zinc finger 3 (Gli3) [22,23,24] (see Figure 2B). Cell lineage tracing experiments have shown that as interzone cavitation and joint capsule formation occurs, articular cartilage and other synovial joint components are originated specifically from this interzone population of cells [24,25,26]. Notably, Hyde et al. [26], have shown that, unlike the remainder of chondrocytes in the cartilage anlagen, articular chondrocytes are derived from a specific group of chondrocytes, which have never expressed matrilin-1 (Matn1). In contrast, pre-chondrocytes found at secondary ossification sites at the edge of cartilage entities are instead transient in nature, proliferating and increasing in size before undergoing hypertrophy and contributing to bone growth [22]. Hypertrophic chondrocytes exit the cell cycle, secrete a distinct ECM rich in collagen X, and can be characterised by the expression of terminal markers of hypertrophic differentiation such as runt-related transcription factor 2 (Runx2), matrix metalloprotease 13 (MMP13), Indian hedgehog (Ihh), and collagen X [27,28,29,30,31]. Mineralization and blood vessel formation are also hallmarks of the phenotype [32]. Until recently, it was thought that the hypertrophic chondrocytes eventually undergo apoptosis allowing the infiltration of osteoblasts and bone formation, however, new evidence suggests some hypertrophic chondrocytes can survive the transition and become osteogenic cells [33]. Although evidence suggests that articular chondrocytes have a distinct embryonic origin from those that form the growth plate, under pathological conditions such as osteoarthritis (OA), chondrocyte hypertrophy is reactivated leading to calcification and outgrowths of bone, indicating that permanent articular chondrocytes can also acquire features of a more transient phenotype [34,35]. Chondrocyte hypertrophy is also a common outcome of in vitro and in vivo cartilage TE/regeneration strategies [30,36,37,38,39,40,41]. TE strategies must therefore focus on creating ‘permanent’ cartilage, which is able to maintain its structure and function throughout life and withstand hypertrophic differentiation [42] (see Figure 2A). A clearer understanding of the mechanisms and factors that trigger and suppress entry into the hypertrophic differentiation pathway will therefore be key to the success of future approaches.

### 2.2. Disease and Trauma

The low cell density and avascular nature of articular cartilage means that the tissue has limited potential for regeneration and repair following injury [3,43]. Instead, when a traumatic lesion, or degenerative disease occurs, the defect is filled with fibrous tissue, which is often unable to withstand the compressive and shear forces which act upon the joint, leading to cartilage breakdown, pain and immobility (as reviewed [3,43]). Cartilage damage can occur as a result of traumatic injuries such as ligament tears, impact, joint dislocation, infection and inflammation. While biomechanical loading is necessary for articular cartilage homeostasis, abnormal, or altered loading is associated with inflammation, metabolic imbalances and joint instability (see [44]). Damage can also occur gradually as the result of degenerative joint diseases such as OA, characterised by pain, structural changes, gradual loss of articular cartilage, and eventual direct bone-bone contact and joint destruction [45]. In OA it is still unclear whether it is the chondrocytes that drive cartilage pathology, or whether they are just responding to the disease process from elsewhere in the joint [46]; the balance between these processes may differ between patients and disease subgroups. OA is an epidemic problem and a major cause of decreased quality of life in adults [47]. It is estimated that one third of people over the age of 45 have sought treatment for OA in the UK [48], and this number is expected to rise in line with the ageing population and increasing obesity problem [49]. Therefore, there is a great need for the development of new approaches to prevent and treat cartilage damage.

### 2.3. Current Therapies

While knee-replacement surgery is becoming an increasingly common procedure, with around 97,000 replacements carried out each year in the UK alone [50], treatment of cartilage injury at an earlier stage to postpone or avoid total joint replacement would be preferable. Current treatments used in the clinic for cartilage damage include mosaicplasty, autologous chondrocyte implantation (ACI), and microfracture, each of which have shown various degrees of success, but which prove typically unsatisfactory in the long term [51,52]. For example, in the case of mosaicplasty, damaged cartilage is replaced with cartilage plugs (allo- or auto-grafts), which are poor at integrating with the host tissue, and in the case of allografts, have the potential for disease transmission [53]. ACI was first described in 1994, and involves the use of autologous chondrocytes, which are isolated from an uninjured area of the knee, expanded, and injected into the area of the defect [54]. The procedure has since been adapted to include the use of collagen/hyaluronan (HA) scaffolds (matrix-induced ACI (MACI)), allowing for improved cell attachment and outcomes [55,56]. However, numerous limitations bedevil ACI/MACI outcomes, including donor site morbidity, the amount of time taken to expand the cells ex vivo, the inability to treat large cartilage defects, expense, and patient disability for long periods of time before surgical implantation can take place [51,57]. In addition, adult chondrocytes are known to de-differentiate during the expansion process, losing their spherical morphology and ability to synthesize aggrecan and collagen II [58,59]. Following implantation, hypertrophic growth has also been associated with these grafts [60]. Chondrocytes of older patients also have age-related intrinsic changes, such as decreased mitotic activity and telomere length [61,62]. As already mentioned, it is also unclear whether degenerative diseases such as OA are an ageing disorder of the chondrocyte itself, or whether chondrocytes are simply responding to the disease process initiated elsewhere [46]. Ultimately, therapeutic options for articular cartilage repair remain insufficient, despite the increasing prevalence of cartilage disease. While the use of chondrocytes in ACI/MACI procedures has marked the first steps in repairing damaged cartilage, with an ageing population the need for a more reliable, efficient and durable method of cartilage repair is clear. 

## 3. Mesenchymal Stem Cells

Stem cells have become an attractive alternative to the use of chondrocytes for cartilage TE strategies, due to their relative abundance, self-renewal, and multipotent or pluripotent capabilities, therefore avoiding many of the limitations of chondrocytes [63,64,65]. The best stem cell source for cartilage TE is yet to be identified, with MSCs, embryonic stem cells (ESCs) and induced pluripotent stem cells (iPSCs), all being considered due to their individual strengths and weaknesses (as reviewed [66]). Ease of isolation, differentiation potential, surface marker expression and cost are just some of the factors that must be considered. An obvious benefit of ESCs and iPSCs is their therapeutic flexibility and continuous self-renewal capabilities. However, the use of ESCs carries high ethical and political concerns, the possible need for immunosuppressants, and the issue of tumorigenicity [67,68,69]. iPSCs also carry a high risk of teratoma formation, show low reprogramming efficiencies, and have been found to retain the epigenetic memory of their somatic cell of origin [70,71,72]. While the use of ESCs and iPSCs may become particularly important for the regeneration of tissues that cannot be repaired by alternative cell types, their limitations have meant that human MSCs (hMSCs) have remained a particularly attractive cell source for cartilage (and other) TE strategies [73,74]. hMSCs are relatively easily available, can be extracted from multiple tissue sources in relatively large quantities with limited morbidity, are capable of self-renewal, lack tumorigenicity, are applicable to autologous transplantation procedures, and can differentiate into multiple cell lineages, including chondrocytes [75,76,77]. MSCs are also known to secrete soluble cytokines and growth factors in a paracrine fashion, which are thought to add to their therapeutic effects [78]. In addition, MSCs have been indicated to have stronger immunomodulatory properties compared to other stem cell sources, and have been shown to suppress T cell, B cell, dendritic cell and natural killer cell activity [75,79,80,81,82]. MSCs are therefore not only attractive for tissue regeneration via their multilineage differentiation potential, but also for treatment of autoimmune diseases such rheumatoid arthritis (RA), and to help bypass graft-versus-host disease (GVHD) [83,84,85,86]. In addition, MSCs have the obvious benefit over ESCs and iPSCs for cartilage TE, that they are already committed to mesoderm lineages [87], and therefore require less in vitro programming.

hMSCs were first used in a clinical trial to treat full thickness cartilage defects in 2004 [88], and have since been included in an increasing number of clinical trials [89,90,91,92]. Generally, results suggest that hMSCs are a promising cell source for cartilage repair, but there is a general lack of comparative studies and systematic reviews, and much remains to be investigated and optimised (see [73]). Some of these issues have been outlined below.

### 3.1. Isolation and Characterisation of MSCs

Adult MSCs are derived from the mesoderm and reside in the adult body throughout life, generally decreasing in abundance with age [87,93,94]. MSCs were first isolated from bone marrow [95,96], but have since been extracted from numerous sources including, adipose tissue [97], skeletal muscle [65], the synovium [98], and the umbilical cord [99]. The inherent differences among the microenvironments of each of these stem cell populations means that although each share similar phenotypic and functional properties, differences in differentiation capacities, and surface marker expression do exist [100,101]. This review will focus on bone marrow-derived MSCs (BMMSCs), which are the most well characterised and commonly used adult stem cell source for cartilage TE [102]. However, it is important to note that synovial membrane derived MSCs (SMSCs) are also becoming an interesting alternative due to their proximity to articular cartilage and tissue-specific properties for connective tissue repair [103,104,105]. Adipose derived MSCs are also gaining increased interest due to their abundance (5% of nucleated cells versus 0.0001–0.01% for BMMSCs) and the ease with which they can be harvested [106,107].

BMMSCs are isolated from the mononuclear layer of the bone marrow and are characterised by their fibroblast-like morphology, proliferation to form loose colonies of spindle shaped cells, and ability to adhere to tissue culture plastic [108,109]. The cells are passaged when they reach 80–90% confluence, with serial passages and washes generally thought to remove any non-MSC types. However, it is important to note that the resulting cultures are still heterogeneous and this will be discussed in the following section.

Phenotypically, no specific markers for hMSCs have been identified to date. Instead characterisation is based upon a panel of positive and negative markers. Generally, it is considered that hBMMSCs are negative for the hematopoietic markers CD45, CD34, CD14 and CD11, as well as for the co-stimulatory markers CD80, CD86 and CD40, and the adhesion molecules CD31, CD18 and CD56 [80]. The cells are also characterised as being positive for CD105, CD73, CD44, CD90, CD71 and Stro-1, as well as for the adhesion molecules CD106, CD166, ICAM-1 and CD29. Importantly, MSC surface markers have been shown to vary between species, tissue sources, and methods of isolation and culture. For example, a study by De Ugarte et al. [110], noted differences in expression of the cell adhesion molecules CD49d, CD54, CD34, and CD106, between MSCs isolated from bone marrow and adipose tissue. This highlights the need for a more uniform characterisation of MSCs, as it is becoming increasingly difficult to compare and contrast findings between investigators who are likely to be looking at distinct/different cell populations. A further way to identify MSCs is through their multilineage potential to differentiate into adipocytes, chondrocytes and osteoblasts [111]. However, the plasticity of MSCs has also come under further investigation, as studies have shown that the cells are also capable of differentiating down the myogenic and neuronal lineages [112,113,114,115].

In 2006 the Mesenchymal and Tissue Stem Cell Committee of the International Society for Cellular Therapy (ISCT) decided to try and address the inconsistent use of defining characteristics, and lack of universally accepted requirements to define MSCs [111]. It was proposed that the minimal requirements to define hMSCs would be: (i) the ability to adhere to culture tissue plastic; (ii) to express CD105, CD73 and CD90; (iii) to lack expression of CD45, CD34, CD14 or CD11b, CD79α or CD19, and HLA-DR surface markers; (iv) and to differentiate into osteoblasts, adipocytes and chondrocytes. Although these guidelines have provided the first steps in streamlining the characterisation of hMSCs, further refinements are clearly required if their full therapeutic potential is to be met [116,117,118,119,120]. Ultimately, the ubiquitous term of ‘MSCs’ remains one of the most undefined and controversial areas in stem cell research. It has even been suggested that this terminology should be abandoned altogether, with the argument that ‘MSCs’ with identical differentiation capacities do not exist [121].

### 3.2. MSC Heterogeneity

The low frequency of MSCs within human tissue means that the cells require extensive ex vivo expansion before they can be used for in vitro/in vivo testing, or indeed for potential clinical application. However differences in isolation methods, culture conditions and media additives used between different laboratories means that variability in cell yield and the phenotype of the expanded cell products are inevitable [122,123]. As well as improved characterisation of MSCs, standardisation of the procedures used for their isolation and expansion should therefore also be a priority, so that comparisons between studies can be made more effectively. MSCs are known to display a high degree of heterogeneity, and outgrowth of certain subsets of cells driven by the differences in culture conditions is likely to promote or inhibit their differentiation potential [124]. A better understanding of how this is controlled, and which conditions promote the differentiation of MSCs into certain cell types, is clearly needed. In the case of cartilage, better understanding of what drives permanent articular-cartilage formation, rather than fibrocartilage or hypertrophic differentiation is of the upmost importance to their success in TE/regeneration strategies [125,126,127]. Torensma et al. [124] suggest that the leading cause of heterogeneity within MSC cultures is, unsurprisingly, the tissue source, followed by the culture methodology, and then individual donor variation. Remarkably, in the same study, it was also shown that expanded cells which were then frozen and distributed to different laboratories to be grown for one passage, also developed some heterogeneity; indicating that cell culture location also has an effect.

### 3.3. In Vitro Chondrogenic Differentiation of MSCs

The most common and established method for in vitro chondrogenic differentiation of BMMSCs is often referred to as pellet culture and was developed by Johnstone et al. [36] using rabbit BMMSCs. The group then went on to use the same technique with hBMMSCs [37]. Briefly the technique involves spinning 200,000 cells at 500 g in 15 mL polypropylene conical tubes to form spherical cell aggregates. The spheroids are cultured in defined, serum-free medium consisting of high glucose Dulbecco’s Modified Eagle’s Medium (DMEM) supplemented with transforming growth factor beta 1 (TGFβ1), ascorbate-2-phosphate, sodium selenite, transferrin, dexamethasone, insulin, and sodium pyruvate. The technique is highly reproducible, with cell aggregates being harvested at time points up to 28 days, and some groups adapting the protocol to use up to 500,000 cells per aggregate [59]. Mackay et al. [128] went on to show that the addition of TGFβ3 rather than TGFβ1 better supports chondrogenesis. 

The simplicity of the pellet culture method means it has remained popular for examining the various signalling pathways and soluble factors involved in chondrogenic differentiation, as well as for disease modelling [129,130,131]. However, mass transport limitations mean that the pellet cultures are not made up of a homogenous population of cells, and ECM production is not as extensive as you would hope for in an articular cartilage-like tissue, especially towards the centre of constructs [132]. In addition, the spheroid geometry of the pellet culture does not match the structured organisation of cartilage in vivo [7,8].

Murdoch et al. [41], modified the pellet culture method, introducing a porous membrane as a support for the cells. The method uses the same media and supplements as mentioned above, but the cells (500,000) are seeded onto Transwell filters and spun in a 24-well plate at 200 g, creating a shallow multilayer of MSCs that are then able to grow and differentiate into a disc of cartilage-like tissue [41,59,133]. The Transwell culture method has been shown to lead to more rapid and efficient differentiation of MSCs, and the deposition of a more extensive cartilage-like ECM when compared to the pellet culture system [41]. In addition there is a more uniform differentiation of the MSCs with improved mass transport within the shallow and permeable disc geometry [59]. Recently, it has also been shown that the Transwell system allows for the production and assembly of organised and cross-linked collagen networks; helping to explain the robust, flexible nature of the constructs that are formed [133]. However, despite the clear improvements in this technique, many researchers are still using the pellet culture method, perhaps due to the difficulty in reproducing the Transwell method effectively.

These difficulties and limitations have meant that the use of scaffolds/biomaterials to improve the in vitro chondrogenic differentiation of MSCs has become a popular avenue of research, allowing for a better recapitulation of the in vivo environment and production of larger scale cartilaginous constructs that are more clinically relevant. Ultimately though, despite much progress over the last two decades, current methods for the in vitro chondrogenic differentiation of hMSCs are still a long way off reaching the clinic. Indeed, as far as we are aware, pre-differentiated MSCs have not yet been used in any clinical trials for cartilage repair. Perhaps the most pressing concern/limitation regarding the application of pre-differentiated hMSCs is that of chondrocyte hypertrophy, with most differentiation protocols resulting in the formation of chondrocytes with endochondral-like maturation properties rather than a true articular cartilage-like phenotype [36,37,38,41,134,135,136,137]. In contrast, the use of undifferentiated hMSCs for cartilage repair has progressed further, with around 50 clinical trials currently registered at https://www.clinicaltrial.gov/. Published results have generally been promising, and the use of allogenic umbilical cord MSCs have already been approved by the Korean FDA for OA treatment [92,126,138,139,140,141,142,143,144]. However, the use of these cells for cartilage formation is still in its infancy, and from the evidence that is available, protocols to improve the quality of the regenerated cartilage are still clearly required [88,126,145,146,147]. As such, there is an overriding necessity to better understand the mechanisms controlling permanent articular cartilage formation, alongside further evaluation of ongoing clinical trials, before these cells can be used to their fullest potential in the clinic. Whether the greatest success will be seen with the in vitro chondrogenic differentiation of hMSCs, in vivo delivery of undifferentiated cells, or with cell-free approaches that instead harness the body’s own MSCs for repair, remains to be seen. However, what is clear is that the use of current in vitro models (such as the pellet culture) to screen potential bioactive factors (such as growth factors) and culture conditions, as well as investigation into biomaterials that can help support articular cartilage formation, should be a priority, and will be of the upmost importance for the improved development of both in vivo and in vitro regeneration strategies.

## 4. Growth Factors Involved in the Chondrogenic Differentiation of MSCs

The differentiation of MSCs into chondrocytes involves the activation and suppression of a number of signalling pathways and growth factors, with members of the TGFβ, fibroblast growth factor (FGF), and insulin-like growth factor (IGF) families, among others, having all been implicated in the process (as reviewed in Table 1). The TGFβ superfamily has been the most researched of all stimulating factors in the area of chondrogenesis, and is described in more detail in the sections below.

### 4.1. Transforming Growth Factor Beta (TGFβ) Superfamily

The TGFβ superfamily of secreted factors is made up of more than 30 members that can be phylogenetically split into two main groups, namely the TGFβ/Activin and the BMP/GDF sub-families [212,213]. Members of the family are synthesised as large precursor (inactive) molecules, which then undergo proteolytic cleavage following dimerisation, to yield active, mature dimers [214]. All mature monomers of the family share a conserved cysteine knot structure, formed from six characteristically spaced disulfide-bonded cysteines within the C-terminal region [215,216]. In addition, with the exception of GDF3 and GDF9, members of the family contain a seventh conserved cysteine residue, which is required for the covalent linking of the dimeric structures [217]. Upon secretion, the active mature dimers are then able to bind to their respective receptors to initiate downstream signalling, although a number of further regulatory mechanisms exist, such as the secreted antagonists Noggin and Chordin [218], and where ECM molecules such as heparan sulfate (HS) also play a role [169]. In addition, it has been shown for some TGFβ superfamily members (such as TGFβ1/2/3 and GDF8), that the mature ligand is secreted from cells in a large complex which includes the cleaved pro-region, known as the latency-associated protein (LAP) (see [213,219]). These complexes have also been shown to bind to latent TGFβ-binding proteins (LTBPs), which are structurally related to fibrillins and allow for ECM localisation [220]. The complexes remain inactive in the ECM, until the mature ligand is released from the complex by activators such as integrin receptors, proteases, or reactive oxygen species [220,221,222,223,224,225]; thereby adding another level of control to ligand activity.

**Figure 3:**
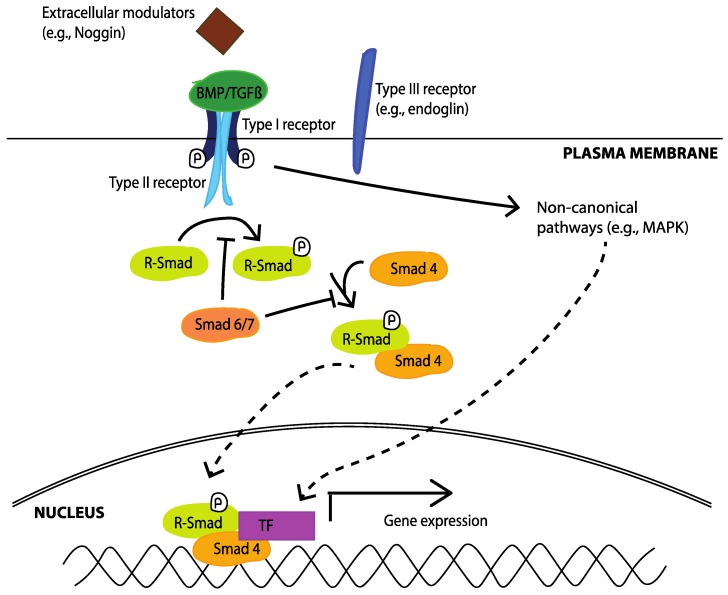
Schematic overview of TGFβ signalling. Binding of a TGFβ/BMP ligand to specific cell surface receptors induces the formation of a heteromeric type II/type I receptor complex. This binding is further regulated by type III receptors/co-receptors. Upon ligand binding, constitutively active type II receptors activate type I receptors. This then leads to the phosphorylation of R-Smads (Smad 2/3 for the TGFβ subfamily; Smad1/5/8 for the BMP subfamily). R-Smads then form heterodimeric complexes with Smad 4 (Co-Smad) and translocate to the nucleus, where they regulate gene expression through interaction with transcription factors (TFs). I-Smads (Smad 6/7) inhibit receptor activation of R-Smads. Besides the canonical Smad signalling pathway, non-Smad pathways, such as the MAPK pathways have also been implicated in TGFβ signalling. Figure adapted from [227].

As extensively reviewed [137,226,227,228], upon binding of a TGFβ superfamily member to its receptor, the formation of a heterodimeric serine/threonine kinase complex is induced, which is composed of a pair of high affinity type I and type II receptors (see Table 2 and Figure 3). In addition, there are a number of type III co-receptors, able to indirectly regulate signalling by binding to TGFβ ligands and modulating their subsequent binding to type I and type II receptors. The type II serine/threonine kinases are constitutively active, and upon ligand binding undergo a conformational change, which leads to the recruitment and phosphorylation of the appropriate type I receptors. Type I receptors then specifically recognise and phosphorylate receptor-regulated Smad proteins (R-Smads). In this regard, there are two classes of R-Smads: the TGFβ responsive Smads, which include Smad 2 and 3, and the BMP-responsive Smads that include Smad 1, 5, and 8. Upon activation, R-Smads form a complex with the common-partner Smad (Co-Smad; Smad 4), and translocate to the nucleus to regulate the transcription of a multitude of target genes. Inhibitory Smads (I-Smads; Smad6/7) can also inhibit the receptor activation of R-Smads by mediating the degradation of receptors and R-Smads. While Smad 7 inhibits all TGFβ superfamily members, Smad 6 is more specific towards the BMP subfamily [229].

Aside from canonical Smad-mediated TGFβ superfamily signalling, other signalling pathways, such as the mitogen activated-protein kinase (MAPK) pathways have also been implicated in the process (reviewed [230]). These non-Smad-mediated pathways can either be directly activated by TGFβ ligands, or can modulate the activity of TGFβ-induced Smad signalling, allowing for crosstalk and modification/fine-tuning of initial Smad-mediated signals.

#### 4.1.1. TGFβ Subfamily

TGFβ is crucial for cartilage maintenance and integrity, as highlighted by mutations in these genes/proteins, which have been shown to lead to OA-like pathology [231,232]. Lack of TGFβ signalling is also reported in aged mice, and murine models of OA [233,234]. This has led to the majority of in vitro chondrogenic differentiation protocols including supplementation with either TGFβ1 or TGFβ3, of which TGFβ3 has been shown to support chondrogenesis more efficiently [36,37,38,39,40,41,59,75,128,133,165]. It has generally been considered that TGFβ is the only well-established inducer of chondrogenesis, leading to the deposition of a matrix rich in PG and collagen II. However, TGFβ-induced chondrogenic differentiation of MSCs is also clearly accompanied by the expression of unwanted hypertrophic markers such as collagen X and MMP13 [36,37,39,41,75,128,165]. In addition, the ectopic transplantation of TGFβ-differentiated MSCs into the subcutaneous pouches of severe combined immunodifficient (SCID) mice has been shown to result in matrix calcification and vascular invasion [38]. Human articular chondrocytes have also been shown to be directed towards hypertrophy when expanded in the presence of TGFβ1 [235]. Interestingly, it has been reported that MSCs differentiated in the presence of TGFβ1 had significantly less mineralisation than those cultured with TGFβ3 [236]. However, taken together, these drawbacks have indicated that further refinement of chondrogenic differentiation protocols are required; as such, researchers are now looking into the use of other/combinations of chondrogenic factors, which can induce the differentiation of MSCs into a permanent articular chondrocyte-like phenotype, and that can withstand hypertrophy. For example, it has been indicated that PTHrP may be able to suppress the hypertrophic effects of TGFβ treatment, without affecting the deposition of a cartilaginous matrix [39,153,208,209]. The use of a wide range of BMP subfamily members are also being increasingly investigated (see Section 4.1.2). Importantly, mutations which lead to elevated TGFβ activity have also been associated with increased bone mass and ossification [237,238], and TGFβ supplementation has been shown to stimulate osteophyte formation in the murine knee joint [239]. This therefore highlights that TGFβ activity is not limited to the articular cartilage tissue of synovial joints, and may well help explain why TGFβ stimulation also leads to chondrocyte hypertrophy and mineralisation (i.e., if MSCs are not first primed towards the articular cartilage pathway). The medium formulations (including growth factors) that are currently used for inducing chondrogenic differentiation of MSCs are potentially overly simplistic, since the formation of permanent articular cartilage is likely to be dependent upon the cross-talk from multiple signalling pathways [20,240]. Investigation into the effects of other chondrogenic factors has therefore become vital.

#### 4.1.2. BMP Subfamily

The unsatisfactory results from in vitro chondrogenic protocols using TGFβ supplementation has led to the extensive investigation of the BMP subfamily of the TGFβ superfamily [153,183,184,185,186,241,242,243]. BMPs have been shown to play a number of essential roles in endochondral ossification, and are important for chondrocyte proliferation and differentiation, helping to maintain the expression of the chondrogenic transcription factor Sox9 [244,245]. Dual knockdown of BMP2 and BMP4 in mice has been shown to lead to abnormalities in chondrogenic condensations, and severe disorganisation of chondrocytes within the growth plate [246,247]. BMP7 is also thought to play a particularly important role in articular cartilage maintenance, with intra-articular injections of the ligand being shown to delay cartilage degradation in mice [248]. In vitro studies have demonstrated that BMPs (predominantly BMP2/4/6/7) can also induce the chondrogenic differentiation of MSCs, and that the use of BMPs in combination with TGFβ is more effective at inducing chondrogenesis than TGFβ treatment alone [183,184,185,186,241,242]; however, the majority of these protocols have also led to extensive hypertrophic differentiation. In contrast, Weiss et al. [153] have suggested that BMP2/4/6/7 and IGF1 were individually not sufficient to induce chondrogenesis of MSCs and that instead TGFβ was also required. Again, when these growth factors were used in combination with TGFβ, collagen X expression was still observed. Caron et al. [186] also looked at the effects of both BMP2 and BMP7 in combination with TGFβ3, and found that while BMP2 promoted chondrocyte hypertrophy, BMP7 inhibited this terminal differentiation. Handorf and Li [243], have looked at varying growth factor requirements throughout the differentiation protocol, evaluating if MSCs may have differing requirements depending on their stage of differentiation. While sequential administration of TGFβ1 and BMP7 did not enhance chondrogenesis to a greater extent than treatment with both growth factors at every feed, the hypertrophic phenotype was significantly reduced; but, while these results were promising, additional reductions in hypertrophy were still thought to be required. This therefore further highlights that examination of factors that can repress hypertrophy, or prime MSCs early on towards a phenotypically stable articular cartilage state, is of profound importance. The identification of such a factor, and its use in combination/sequence with other TGFβ and BMP treatments may be key to generating a permanent hyaline cartilage tissue.

##### 4.1.2.1. GDF5

GDF5, also known as BMP14 or cartilage derived morphogenetic protein 1 (CDMP1), is a particularly interesting member of the BMP subfamily. It is synthesised as a 501 amino acid preprotein (UNIPROT accession number: P43026) [249]. Upon cleavage of the signal sequence (27 residues), the proregion (70 kDa) is proteolytically removed, leaving a 13.5 kDa (120 amino acid) monomer, which will then go on to form a disulfide linked homodimer, or a heterodimer with another TGFβ superfamily member [250,251,252]. GDF5 is well established as playing a critical role in joint development and maintenance [23,171,172,173,253,254,255,256]. Its importance was first highlighted in mice carrying the brachypodism (bp) mutation, which results in changes to the length and number of bones in the limbs, and was found to be the result of mutations in the GDF5 gene [253]; GDF5 was also shown to be highly expressed in the joint interzone, and that mice lacking both GDF5 and GDF6 have further wide spread joint defects and skeletal growth retardation [257,258]; highlighting its importance in the formation of synovial joints. Importantly, GDF5 has also been shown to be required for proper joint formation and homeostasis in humans, and is predominantly expressed in areas of cartilage formation during embryonic development [171,259,260,261,262,263]. Loss of function mutations in the human GDF5 gene have been shown to result in a number of chondrodysplasias such as Grebe and Hunter-Thompson syndromes [259,263], and a single nucleotide polymorphism in the 5′UTR of human GDF5 has also been linked to OA susceptibility [261]. In contrast, overexpression of GDF5 has been shown to enhance chondrogenesis, increase the length and width of bones, and lead to joint fusions [260,262].

Despite the clear importance of GDF5 for skeletal formation, its use for chondrogenic in vitro differentiation protocols and regeneration strategies has been somewhat under-researched compared to other TGFβ superfamily members. However, previous work comparing the effects of TGFβ1 and GDF5 in fetal hMSCs via histological staining, has demonstrated that while TGFβ1 was more stimulatory in terms of GAG production, the combination of both TGFβ1 and GDF5 was synergistic [264]. In contrast, Feng et al. [176] indicated that compared to TGFβ1 (10 ng/mL), GDF5 (100 ng/mL) had a much greater effect on the chondrogenic differentiation of adipose derived rat MSCs, although collagen X levels appeared similar in both TGFβ and GDF5 treated pellets. Interestingly, MSCs transfected with GDF5 and implanted into full thickness articular cartilage defects in the knee joints of rabbits have shown promising results; demonstrating superior repair of hyaline cartilage compared to MSC implantation alone [265]. In addition, most crucially, Zhang et al. [177], have reported that GDF5 (100 ng/mL) inhibited hBMMSCs from expressing collagen X, while promoting the deposition of a cartilage-like matrix. Although their results were not compared to the more commonly used TGFβ, this study has sparked interest in GDF5 as a target for cartilage TE/repair strategies.

**Figure 4:**
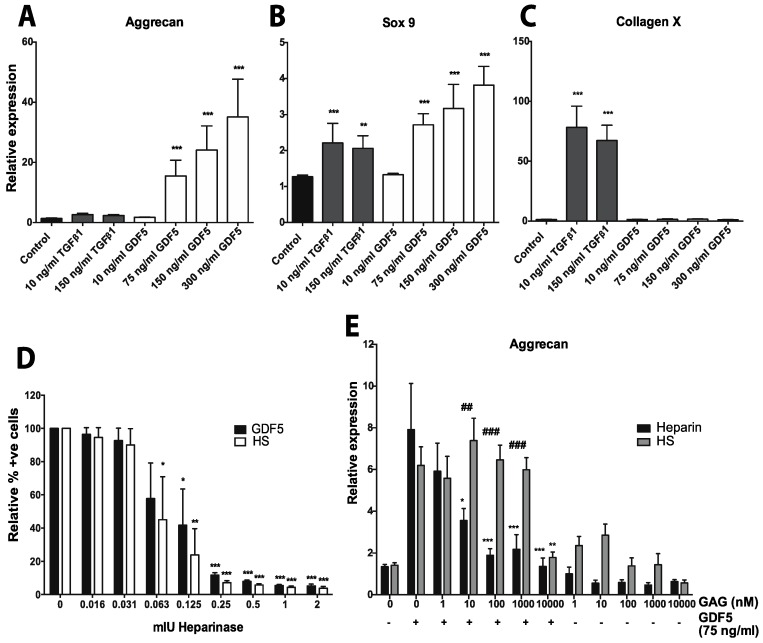
GDF5 promotes the chondrogenic differentiation of hMSCs without inducing hypertrophy, and its activity is modulated by GAGs. GDF5 promotes the expression of aggrecan (**A**) and Sox 9 (**B**), both markers associated with chondrogenesis and ECM production, in hMSC-derived chondrogenic pellets, but importantly, does this without inducing collagen X expression; (**C**), a marker of chondrocyte hypertrophy. The removal of endogenous HSPGs (HS) from the cell surface (by using heparinase) is positively correlated with the reduced level of GDF5 able to bind to the cell surface; (**D**), Exogenous heparin, but not equivalent doses of HS, inhibit GDF5-induced chondrogenic differentiation of hMSCs as monitored by aggrecan expression (**E**), * *p* < 0.05, ** *p* < 0.01, *** *p* < 0.001 versus no addition control; ## *p* < 0.01, ### *p* < 0.001, comparing heparin and HS of same dose (see [266] for full experimental details).

This is exemplified by our own recent work, showing that GDF5 can induce the chondrogenic differentiation of hMSCs, while overcoming the hurdle of hypertrophy [266]. In contrast to TGFβ1, we found that GDF5 induced aggrecan and Sox9 expression (both markers associated with chondrogenesis and ECM production [267]), without increasing the expression of collagen X (the major marker of chondrocyte hypertrophy [268]); see Figure 4A–C [266]. Our data suggests that GDF5 could be used to generate a clinically useful cartilage matrix with a high PG content, while maintaining the chondrocytes in a mature articular cartilage phenotype. As well as building on the results of Zhang et al. [177], our research [266] also complements several other studies published over the past few years [175,179,181,269]. For example, work in an OA rat model has demonstrated that GDF5 is expressed in healthy pre-hypertrophic cartilage, but is not evident as OA develops [175]. A further study with human chondrocytes has also shown that GDF5 stimulation promoted the expression of aggrecan, while inhibiting collagen X expression [179]. Consistent with what we observed, Murphy et al. [181] demonstrated that collagen X expression was significantly increased in the presence of TGFβ1 but not GDF5, i.e., in 7-day hMSC-derived chondrocyte pellets. However, our results are in contrast to another recent study suggesting that GDF5 can promote the hypertrophy of hMSC-derived chondrocyte pellets [180]. This work, however, looked at the effect of GDF5 in combination with TGFβ3, and not in isolation. We have demonstrated that GDF5 alone does not increase collagen X expression, and that the combination of GDF5 and TGFβ1 is no more potent at inducing collagen X than TGFβ1 treatment alone [266]. 

Thus, overall, there is increasing evidence supporting the potential use of GDF5 in cartilage TE strategies, especially when this growth factor is supplied to hMSCs in the absence of TGFβ. Future studies to look at the expression of a wider repertoire of genes involved in chondrogenesis, as well as additional biochemical assays (for example to quantify PG content) would help to further determine the effects of GDF5 on the chondrogenic differentiation of hMSCs. A recent study in human umbilical cord perivascular stem cell-derived chondrocyte pellets demonstrated that GDF5 enhanced proliferation, but had no effect on the expression of chondrogenic-related genes [270], therefore indicating that the effect of GDF5 may be specific to the source of stem/stromal cells. 

Importantly, the supplementation of hMSCs with GDF5 rather than TGFβ1/3 may provide an effective way to achieve the aim of forming hyaline rather than hypertrophic chondrocytes from hMSCs, and strongly suggests that a transition to using GDF5 in hMSC-based cartilage engineering strategies could help to overcome this long-standing hurdle [266]. However, hMSC heterogeneity [271], along with the inability of being able to form a scalable tissue, need to be overcome if successful clinical implementation is to be achieved. A more robust quality control of cell preparations, that can better predict clinical outcomes, and/or allow for the purification of subpopulations of cells with improved chondrogenic potential, is therefore of the upmost importance (see [272]). The difficulties surrounding the use of hMSCs, has also meant that researchers are now looking into alternative solutions to cell therapy. Conventionally the strategy would be to deliver expanded hMSCs (undifferentiated or differentiated) to the repair site, but recent work has led to the opinion that the beneficial effects of hMSCs (or other stem cells) for tissue regeneration are not only due to cell restoration (and engraftment), but can also be attributed to the trophic factors that hMSCs release (see reviews [273,274]). As a result, research is now being directed into the identification and delivery of paracrine factors to the injury site, which can then modulate the environment and evoke a repair response from the resident cells [275,276,277,278,279]. These cell free approaches to tissue regeneration are exciting; e.g., overcoming the issues of cell sourcing, expansion and differentiation, as well as the strict regulatory issues that surround cell therapy. However, they come with other challenges, including the effective and safe delivery and/or controlled release of the bioactive factors [277,280]. These issues, which are relevant to both cell-free and cell-based regeneration strategies, will be explored in further detail within the following sections.

## 5. Glycosaminoglycans

As well as the difficulties in identifying the correct growth factors (and combinations thereof) to target for cartilage TE/regeneration strategies, the inherent instability of these proteins has also hampered their potential use. Growth factors are known to be susceptible to proteolytic degradation, are rapidly cleared from the injury site, and demonstrate burst release pharmokinetics [281,282,283]. Together these factors have largely meant that supraphysiological quantities are required to get anywhere near the desired outcome, resulting in economically unsustainable costs for clinical translation [284,285,286,287]. In addition, the safety of growth factors is still under debate due to the increasing number that are being identified as proto-oncogenic, and this worry is only heightened by the high doses that are currently required [288,289,290,291]. The use of carriers that can reduce the quantity of exogenous growth factors required, through their localisation, protection or enhancement, or indeed that could harness endogenously produced growth factors, would therefore be ideal. Research into the use of GAGs for these approaches is proving particularly promising, and presents an attractive therapeutic opportunity for TE strategies [163,195,292,293,294,295]. 

In animal cells there are an estimated 10^5^–10^6^ copies of HS and chondroitin sulfate (CS) on the cell surface, which range from ~40–160 nm in length; these GAGs are present at mg/mL concentrations in the ECM, highlighting their abundance and importance. Moreover, solution structure studies of sulfated GAGs have indicated that the oligosaccharides are highly dynamic, with a high degree of conformational plasticity (although forming stiffened random coils), allowing them to interact with a diverse number of proteins, and adding to the complexity of the interactions [296]. All GAGs, with the exception of HA (and heparin, once it has been secreted by mast cells), exist as PGs by attaching to serine residues on core proteins [297]; alternatively, keratan sulfate (KS) can be *N*-linked. PGs are produced by virtually all mammalian cells and can be found on the cell surface, within the ECM, or stored in secretory granules [298]. PGs can include only one GAG chain (e.g., decorin), or can have over 100 GAG chains, as is the case for aggrecan, the most predominant PG in cartilage [299,300]. Aggrecan consists of around 100 CS and 30 KS GAG chains extending from a large protein core of approximately 250 kDa [301]. In addition, this PG forms large aggregates (size, 1–4 μm), with hundreds of aggrecan molecules non-covalently bound on a single HA chain, where the interaction is stabilised by cartilage link protein [302]. The biomechanical properties of cartilage are largely due to the high negative charge provided by associated GAG chains, and the large size of the biopolymer, which is held in place by collagen networks. These factors render the PG immobile and unable to redistribute itself, allowing the matrix to become water swollen (Gibbs-Donnan effect), resistant to deformation, and able to resist compressive force [303]. The highly sulfated GAG chains of aggrecan are also responsible for sequestering and modulating the accessibility of molecules, such as growth factors, which bind via positively charged amino acid residues [304]. Other PGs expressed in cartilage include glypican, cell surface syndecans and the large HSPG perlecan [162,305,306,307]. Via its HS chains, perlecan has strong affinity for collagen VI, and can bind to fibronectin and laminin, via both its HS chains and core protein [308]. Through these interactions perlecan has been shown to play an important role in sequestering FGF2 and softening the ECM within the immediate vicinity of the chondrocytes (the pericellular matrix (PCM)), providing an environment suitable for mechanotransduction [161,308].

The physiological and pathological functions of PGs are vast; e.g., helping to define tissue form, and dynamically modulate function and remodelling during health and disease [309,310]. The specific functions of GAGs/PGs include ligand binding/immobilisation/retention, promotion of ligand/receptor interactions, protection of ligands from proteolysis, and facilitation of cell-cell and cell-matrix interactions [156,311,312,313,314,315,316,317,318,319,320]; all of which can have a profound effect on cellular activity. The variable lengths and sulfation patterns of GAG chains on PGs, makes them the most structurally complex and information dense molecules found in nature [309]. Importantly, the intrinsic heterogeneity of sulfated GAGs allows for diverse patterning and multifunctionality, and as such the understanding of how these ‘sugar codes’ control tissue development and homeostasis is of huge therapeutic interest [321,322,323,324]. This is particularly apparent in the case of heparin/HS, where the huge degree of versatility has opened up the concept of ‘heparanomics’, seeking to better understand how HS structures interact with proteins to modulate biological activity [325,326]. It has been estimated that over 1 × 10^6^ structurally different sequences are possible from a just a short HS octasaccharide; emphasising the point that these molecules could potentially accommodate an enormous number of protein-binding epitopes [327]. Research aimed at enabling a better understanding of this complexity and its functional consequences is ongoing [328,329,330], and it is ultimately hoped that this will allow for improved modulation of cellular activity and thus, TE/regeneration strategies.

### Role of GAGs in Stem Cell Differentiation and Development

The extensive work of Merry and colleagues has highlighted the importance of HS and HSPGs in stem cell differentiation and embryonic development [331,332,333,334,335]. For example, the neural differentiation of mouse ESCs (mESCs) expressing green fluorescent protein (GFP) under the control of the neural marker Sox1, has been used to show that, compared to undifferentiated mESCs where HS sulfation was low, GFP-expressing neural progenitor cells demonstrated increased levels of both N- and O-sulfation, as supported by high levels of HS biosynthesis enzymes such as NDST4 and 3OST [331]. Variations in sulfation patterns between mESCs and neural progenitor cells were also confirmed using epitope specific antibodies, which show preferential binding to specific patterns of sulfation in the HS chain [331]. The group also demonstrated that the phage-display antibody RB4EA-12, which specifically binds to 6-O sulfated structures, had significantly increased expression in the Sox1-GFP expressing neural progenitors compared to the undifferentiated mESCs. Further to this, EXT1^−/−^ mESCs (that are unable to form an HS polymer) placed in neural media were unable to differentiate, unless supplemented with soluble heparin. It was also shown that exogenously added GAGs, of particular sulfation patterns, concentrations, and chain lengths were also able to support the neural differentiation of wild type mESCs [334]. 

Taking a similar approach, the Merry group have also characterised changes in HS expression during differentiation of mESCs down the mesoderm/hematopoietic lineage, using a cell line where GFP is under the control of the Bry gene (which is expressed in mesoderm but not in ESCs), alongside a panel of HS sequence specific antibodies [332]. Of particular significance was the antibody HS4C3 which was specifically expressed within the emerging Bry^+^ population, but that disappeared from the cell surface as cells differentiated into mature hematopoietic cells. In vivo findings also demonstrated that HS4C3 binding sequences were restricted to the mesoderm during gastrulation. The rapid turnover of the HS4C3 HS binding sequences at the cell surface during different stages of differentiation indicates how highly regulated and important HS sulfation patterns are during development, allowing different growth factors to bind at specific spatio-temporal locations to instigate specific signalling pathways. Understanding the details of HS sequences that are involved in the formation of various tissues, will therefore lead to a better understanding of how stem cell differentiation and development is regulated and controlled. In a subsequent study, EXT1^−/−^ mESCs were only able to differentiate into hematopoietic lineages with the addition of heparin [333]. In contrast (and in contrast to the previously discussed work in the neural differentiation system), the addition of chemically N- or O-desulfated heparin oligosaccharides, or heparin chains shorter than 18 saccharides, were unable to restore differentiation. Together these results highlight the widespread importance of HS in embryonic tissue development. While the low sulfation patterns seen in mESCs help to keep the cells in a pluripotent, undifferentiated state, dynamic changes in the sulfation patterns of HS appear to be required for growth factor activity and stem cell differentiation.

Consistent with the findings above, Nairn et al., [336] examined transcripts encoding the glycosylation machinery during stem cell differentiation, and demonstrated clear correlations between transcripts and changes in glycan structures. In addition, with more particular reference to the importance of GAGs in cartilage development, bioimaging has been used to demonstrate changes in the staining intensity of CS and HS during the chondrogenic differentiation of ESCs [337]. In addition, homozygous mice lacking either exostosin 1 or 2 (EXT1/EXT2; responsible for the polymerisation of growing HS chains), fail to gastrulate, while heterozygous mutants are likely to develop hereditary multiple exotoses, characterised by abnormal cartilage differentiation, premature hypertrophy and bony outgrowths [338,339]. In addition, Stickens et al. [339] showed that all EXT2^+/−^ mice display abnormalities in cartilage differentiation, with disorganisation of chondrocytes and premature hypertrophy in the cartilage. Similarly, conditional knockdown of EXT1 in limb bud mesenchyme also leads to dysregulation of BMP signalling and delayed chondrogenic differentiation [340]. Furthermore, HS chains on the PG perlecan within the growth plate have been shown to be involved in the binding of FGF2 to its receptors FGFR1–4, leading to the regulation of chondrocyte proliferation and bone growth [162]. Importantly, however, perlecan can only deliver FGF2 to its receptors following the removal of CS; thus the CS chains on perlecan allow for the sequestration of FGF2 within the PCM of cartilage tissue, while the HS chains help with its delivery to receptors [161,162]. FGF2 has also been demonstrated to be liberated from perlecan HS chains upon injury or mechanical compression [341]. In humans, a mutation in 3′-phosphoadenosine 5′-phosphosulfate synthase 1 (PAPSS1; a bi-functional enzyme with both ATP sulfurylase and adenosine 5′-phosphosulfate kinase activity), which introduces a premature stop codon and disrupts GAG sulfation, as well as other mutations that lead to disruption in sulfation patterns, have been shown to result in osteochondrodysplasias (disorders in the development of cartilage/bone) [342]. A study has also shown that there is significantly higher expression of the endosulfatases, Sulf1 and Sulf2, in OA versus normal articular cartilage, indicating that changes in 6-O sulfation patterns post biosynthesis can contribute to cartilage pathology [343]. In addition, Sulf1 and Sulf2 have been shown to modulate cartilage homeostasis via their differential effects on BMP and FGF signalling [344]. Together these studies indicate that cartilage is particularly sensitive to sulfation patterns within GAG sequences, with key signalling pathways being dependent upon particular domain structures and sulfation patterns being present; see Table 1. The predominant role of GAGs and PGs in the formation and maintenance of the ECM of articular cartilage, makes them particularly interesting as factors for improving cartilage TE strategies, and a better understanding and identification of GAG sequences involved in articular cartilage formation and disease is clearly required. 

Exogenously added HS, and heparin have been shown to stimulate cartilage nodule formation and growth in chick limb bud mesenchyme micromass cultures [345]. In contrast, although HS treatment alone does not appear to have an effect on the in vitro chondrogenic differentiation of MSCs, addition in combination with TGFβ3 and BMP2 has been shown to potentiate chondrogenic activity, more so than growth factor treatment alone [346,347]. It has been suggested that this may be due to exogenous HS limiting the ability of ligands to bind to endogenous HSPGs that would usually prevent/regulate their binding to receptors [346]. Consistent with this, overexpression of syndecan-3, a major HSPG expressed during chondrogenesis, was shown to impair the ability of BMP2 to promote chondrogenic differentiation [346]. On the other hand, the inclusion of the HSPG perlecan in biomaterials has been shown to enhance cartilage TE strategies, prolonging the release of BMP2 [348]. Exogenous HS has also been shown to prolong TGFβ1-mediated signalling in hMSCs [169]. In addition, a recent study has also demonstrated that knockdown of perlecan inhibits the chondrogenic and adipogenic differentiation of SMSCs, but not osteogenic differentiation [349]. 

Ultimately, the ECM, and more specifically the PCM, which consists of the cell surface and immediate local ECM, plays a major role in maintaining homeostasis and directing cell behavior [350]. It provides a scaffold for cell attachment and spreading and helps provide the shape and mechanical properties of many tissues, including cartilage [304,351,352,353,354]. GAGs and PGs are key components of the PCM/ECM, keeping the matrix hydrated through their high negative charge, determining the compressive properties of the tissue, modulating morphogen gradients, and controlling the activity of ligands. A greater understanding of GAG structure within target tissues (the composition of which varies considerably with cell type and location), and how these are altered during development and disease, will therefore be of huge importance in the success of future TE strategies [355,356,357]. The elucidation of GAG structure/function relationships has lagged behind that of proteins and nucleic acids, largely because of limitations in the research methods available; e.g., GAGs cannot be amplified against a template, and as such cannot be sequenced in the same manner as DNA/RNA [297]. Adding to the challenge is their vast heterogeneity [321] and the high degree of sequence/conformational flexibility, likely underpinning the diversity and complexity of GAG-ligand interactions [296,328,358]. Despite these challenges, the persistence of the PG community has meant that much more advanced synthesis, modelling and sequencing tools are now becoming available (see [321,326,359,360,361]); in line with these developments, there is the hope that the full potential and full biological significance GAGs can soon be realised.

## 6. Biomaterials

A wide range of materials have been used for cartilage scaffold fabrication, including natural polymers such as collagen, fibrin and HA, as well as synthetic polymers such as polylactic acid (PLA) and polyglycolic acid (PGA), and self-assembling peptides (see [362]). Natural polymers have the obvious advantage of biological recognition, providing the cells with ECM molecules that can positively support cell function and adhesion [363,364,365,366]. Collagen, being a major component of the cartilage ECM, is a widely used natural polymer in cartilage TE, and is the most common scaffold to be incorporated into MACI procedures [55,60,367,368]. However, due to its abundance and versatility, collagen I (rather than type II) is usually used [369]. Collagen scaffolds have also been used in clinical trials immediately after microfracture treatment, although assessment of the benefit of this treatment still requires further analysis [370,371,372]. Multi-layered collagen scaffolds, which can more effectively mimic the zonal structure of articular cartilage, have also been recently examined in large animal models [373,374]. Histological analysis demonstrated that these scaffolds alone (i.e., cell-free) were able to initiate the formation of well-structured osteochondral tissue. Collagen I gels in combination with 2D micro patterning and single cell culture has also been used as a platform to investigate conditions that control chondrocyte de-differentiation [375]. This approach also offers a method from which niches for targeted differentiation of hMSCs could be investigated.

As well as collagen, HA and chitosan have also been extensively studied as natural polymers for cartilage TE/regeneration strategies. The HA-based scaffold, Hyalograft C, demonstrated promising results for the treatment of articular cartilage lesions, and like collagen, has also been used in MACI procedures [376,377]. However, results appeared less convincing for chronic lesions [378], and the product was recently discontinued failing EMA approval. More promisingly, a chitosan-based product, BST-CarGel, has been shown to result in better organised tissue repair compared to microfracture treatment alone [379], and further clinical evaluation will indeed be interesting to follow.

However, a number of concerns over the use of natural polymers, such as the feasibility of producing sufficient amounts of these materials for clinical applications, the degree of reproducibility, and assurance of pathogen removal, has led to a number of synthetic polymers being investigated as alternatives (as reviewed [380]). Synthetic polymers offer the benefits of processing flexibility and mass production, lack of immunological concerns, and greater control over degradation rates (see [381]).

Research involving synthetic polymers for cartilage TE is largely focused around degradable polyesters that have been FDA/EMA approved, such as PGA, PLA and their copolymer, poly (lactic-co-glycolic acid) (PLGA) (see [382,383]). PGA and PLA are degraded into glycolic acid and lactic acid respectively, both of which can be metabolised by the body and removed by natural pathways. A recent study has also indicated that the release of lactic acid from PLA scaffolds can be beneficial to chondrocyte ECM synthesis [384]. PLA contains a methyl side group within each monomer unit, which contributes to its hydrophobicity and slower degradation rate compared to PGA [385]. While the greater stability and high compressive strength of PLA may be beneficial to cartilage TE strategies [386], the hydrophobicity of the polymer limits cell attachment and viability [387]. In contrast to PLA, PGA is a rigid polymer with a high degree of crystallinity [385]; it is also hydrophilic and lacks methyl groups, which contributes to its higher degradation rate. The copolymer PLGA can therefore be used to tailor degradation rates and compressive strength, with altered ratios of PLA and PGA being used to fine-tune these properties. One study looked at the effects of PLGA composition on the cell adhesion and growth of bovine articular chondrocytes [388]; here non-woven PGA meshes were coated with varying quantities of PLA via solvent evaporation to give composites with PLA contents ranging from 0 to 68%. The compressive strength and degradation time was shown to increase with increasing PLA content, with those meshes containing 68% PLA lasting for 45 days with a compressive modulus of 20 kPa, while 0% PLA meshes degraded after just 5 days and had a compressive modulus of 1 kPa; however, the hydrophobic PLA was shown to decrease cell seeding efficiency, adhesion and proliferation. PLGA is also considered an expensive synthetic polymer and, as such, the relatively inexpensive polycaprolactone (PCL) is also being widely incorporated into many strategies [389,390,391]. This polymer has also been used to overcome the brittle properties of PLGA and its flexibility means that electrospun PCL nanofibrous sheets can be rolled or moulded over surfaces, e.g., of articular joints; making them excellent candidates for skeletal TE strategies [392]. However, like PLA, the downside of PCL lies in its hydrophobic nature and lack of cell surface recognition sites. Together the relative positive and negative attributes of synthetic polymers means that co-polymerisation of various combinations of both synthetic and natural polymers has become an active area of research [389,391,393,394,395,396,397,398].

### 6.1. Electrospun Scaffolds

As well as choice of polymer, scaffold form is also an important factor to consider. Electrospinning is a popular, simple, and cost-effective technique which allows for the production of both nano- and microfibrous polymer scaffolds (see [399]). Briefly, the technique involves dissolving a polymer in a suitable solvent and feeding it through a metal capillary with a high voltage applied, sending a jet of highly charged molecules in solution towards a collector of opposite charge [400]. As the jet travels, the solvent is evaporated, leaving a mesh of polymer fibers. The applied voltage, working distance, rotation rate, choice of solvent, polymer concentration, and flow rate, are all parameters that can be adjusted to generate scaffolds with different characteristics and fiber morphologies [401]. A number of groups have used electrospun scaffolds for cartilage TE purposes [398,402,403]. Sonomoto et al. [398] demonstrated that their PLGA electrospun nanofibrous scaffolds (a 50:50 ratio of PLA to PGA) could induce the differentiation of hMSCs into chondrocytes, without the addition of any cytokines. Wise et al. [402], have also compared orientated PCL electrospun scaffolds of approximately 500 nm and 3000 nm fiber diameter. The group demonstrated that hMSCs preferentially orientated along the direction of the fibers, and maintained their orientation during chondrogenic differentiation. The nanofibrous scaffolds (500 nm) were shown to enhance chondrogenic differentiation when compared to the microfibrous scaffolds (3000 nm), as indicated by type II collagen and aggrecan expression. 

An issue of electrospinning is the inherently small pore sizes generated (typically < 10 μm), and the low thickness of fibers (typically ranging from 10 to 10,000 nm in diameter; limited by the slow rate of production), which together means that 2D membranes with poor cellular infiltration rather than true 3D scaffolds are often formed [404,405]. As such, techniques such as multilayering, or the incorporation of hydrogels are being investigated [395,406,407,408,409]. Indeed, the native ECM consists of a fibrous protein network infiltrated with PGs, and so inclusion of a hydrogel within electrospun fibers seems a natural step in creating an ECM-mimetic with good levels of hydration, cell infiltration and the potential for controlled release of bioactive factors. A technique involving the combination of electrospinning and electrospraying allows for the rapid fabrication of fiber/hydrogel hybrid scaffolds with improved and more uniform cell infiltration [407,409]. Chen et al. [395] have also recently designed a porous 3D scaffold using electrospun gelatin/PLA nanofibers crosslinked with HA. Chondrocytes cultured in the scaffolds remained viable, and histological staining demonstrated that the construct could enhance the repair of cartilage in vivo. In an alternative approach Coburn et al. [410] have developed a fiber-hydrogel composite, using a novel electrospinning technique, whereby the fibers are spun onto a water coagulation bath (rather than a solid surface). This allows for the formation of a rapid 3D fiber mesh with porosity suitable for hydrogel and cell infusion by means of simple mixing, and which is injectable and allows for homogenous tissue growth. They demonstrated that the density of the fibers within the constructs had an effect on tissue formation, with higher (40% dry weight) fiber density leading to greater MSC proliferation, while lower (10% dry weight) fiber density led to the greatest amount of matrix production following chondrogenesis. However, most characterisation of chondrogenesis in this study seems to have been on fiber-hydrogel composites made from stacking to form a 3D multilayered scaffold, rather than those made by the novel electrospinning technique. Compared to hydrogels alone, the stacked composites showed increased ECM production and greater response to mechanical stimulation, which resulted in a drastic increase in tissue production and near native levels of ECM (although ECM composition was not examined). Owing to the structured composition of articular cartilage, stacked composites, where each layer could be engineered to release different combinations of bioactive factors, may be more effective compared to one homogenous matrix. However, it should be noted that Coburn et al. [410] did not look at any markers of chondrocyte hypertrophy within their study.

### 6.2. Hydrogels

Hydrogels, water swollen hydrophilic polymers that are able to retain large quantities of water, are also popular for cartilage TE strategies, as they allow the formation of an effective 3D environment, can be processed in relatively mild conditions, and can be delivered in a minimally invasive manner (see [411,412]). The high water content of these gels creates a permeable matrix that can effectively mimic the ‘softness’ of cartilage, allowing for easy nutrient transfer, and which can signal to cells through both mechanical and chemical signals [411]. Given these properties, a wide range of both natural and synthetic hydrogels have been investigated for cartilage TE. 

Examples of natural hydrogels frequently used for cartilage TE include fibrin and alginate. Ho et al. [413], compared the chondrogenic differentiation of BMMSCs seeded into fibrin hydrogels or fibrin-alginate composites. MSCs encapsulated within fibrin hydrogels showed increased mesenchymal condensation compared to fibrin-alginate constructs. The fibrin hydrogel encapsulated cells also showed increased chondrogenic differentiation with an increase in expression of collagen type II and aggrecan, and unlike the fibrin-alginate gels, did not appear to undergo hypertrophy. Interestingly though, alginate sulfate hydrogels have been shown to support a chondrocyte phenotype, and by combining alginate sulfate with nanocellulose it is possible to create a printable bioink, which could therefore allow for the spatial deposition of hydrogels with micrometer precision [414].

Nguyen et al. [7], have used different combinations of both synthetic and natural biopolymers to create hydrogels that can direct MSCs to differentiate and form ECM that is characteristic of the superficial, middle, or deep zones of articular cartilage (see Figure 1). For example, the combination of CS and MMP-sensitive peptides incorporated into polyethylene glycol (PEG) hydrogels reportedly led to high levels of type II collagen, low levels of PG expression, and a low compressive modulus characteristic of the superficial zone. In contrast, PEG-HA hydrogels induced low collagen II and high PG levels leading to a high compressive modulus more characteristic to that of the deep zone. In a further study they demonstrated that layer-by-layer organisation of these hydrogels to create a single 3D scaffold can stimulate MSCs to differentiate and form a multilayered ECM similar to that of native articular cartilage [8].

Although hydrogels clearly offer much promise, a common issue is the toxicity problems associated with their chemical crosslinking [415]. A number of groups have therefore been investigating self-assembling peptides, made up of alternating hydrophilic and hydrophobic amino acids, which spontaneously form nanofiber scaffold hydrogels in NaCl solutions [416,417]. These self-assembling peptide hydrogels have the advantages of traditional hydrogels, but also avoid the use of toxic cross-links, harmful degradation products, and undesired immunological responses, making them a very attractive option for TE strategies [416]. Zhou et al. have developed a novel self-assembling peptide hydrogel, which is based around the co-assembly of two aromatic peptide amphiphiles; i.e., a structural peptide, fluorenylmethoxycarbonyl-diphenylalanine (Fmoc-FF) and a functional Fmoc-coupled RGD peptide (arginine-glycine-aspartic acid) (Fmoc-RGD), which corresponds to the cell-binding domain of the common ECM component fibronectin [418]. These short di/tri-peptides were shown to lead to the formation of a simple, cost-effective and bioactive hydrogel, which could be formed at neutral pH and effectively support cell attachment and spreading [418,419]. In addition, the versatility of the hydrogel means that it could also be decorated with other bioactive peptides. Similarly, Saiani et al. [420] have formed simple FEFEFKFK self-assembling octapeptide gels, which can also allow for the addition of bioactive peptides. The gels have been shown to self-assemble into β-sheet structures which can promote the round morphology and ECM production of chondrocytes [421].

Another limitation of hydrogels is that although MSCs undergoing chondrogenesis appear to prefer the softer material of a hydrogel over fibrous scaffolds, hydrogels are limited in their mechanical properties and, thus, this may negatively affect their survival within a joint [410]. As such, the use of fiber/hydrogel composites (as discussed in Section 6.1) is perhaps more likely to succeed in cartilage TE applications. However, considerable variation in the stiffness of self-assembling peptide hydrogels can be achieved through modulating the assembly conditions [422].

### 6.3. GAG Incorporation and Application

As discussed above, synthetic polymers are becoming increasingly popular in TE strategies, due to their reproducibility, non-immunogenic properties, and ability for up-scaled production [381]. However, the bioinert properties of synthetic materials, means that they usually have to be enhanced with additives such as expensive growth factors and cytokines. A more cost effective method of promoting the biofunctionality of synthetic scaffolds is thought to be through the use of GAGs (as discussed in Section 5). 

A number of groups have pioneered the incorporation of GAGs into synthetic polymers, in order to produce functionalised scaffolds that combine the advantages of both synthetic and natural polymeric materials [335,423,424]. The incorporation of GAGs into hydrogels has utilised both covalent and non-covalent conjugation, which allow for the tunable adjustment of matrix characteristics and release of bioactive factors [424]. Kim et al. [425] seeded MSCs into photocrosslinkable HA hydrogels and cultured them with varying levels and durations of TGFβ3. Results demonstrated that relatively short-term exposure (7 days) to a high level of TGFβ3 (100 ng/mL) was more effective at inducing and maintaining cartilage formation when compared to constant delivery of a lower dose (10 ng/mL) over a 9-week period. In agreement with this, Bhakta et al. [189], have reported that heparin containing hydrogels reduced the burst release of BMP2, and sustained its activity, however, it was the initial burst release of BMP2 that was found to be important for optimal bone formation. These results indicate that the controlled degradation of biphasic scaffolds might allow for both the initial release of a high dose of growth factor, followed by prolonged activity [426].

Compared to the incorporation of GAGs into hydrogels, their immobilisation onto surfaces such as electrospun fibrous scaffolds has proved a more difficult task. Typically GAGs are anchored via covalent immobilisation, however, this has been shown to restrict the conformation of the bound GAGs and their functionality [395,427,428,429]; e.g., in some situations the oligosaccharides are required to be internalised with signalling receptors [430,431,432]. Mahoney et al. [433] have developed a method for the non-covalent immobilisation of heparin to surfaces, which involves the use of cold plasma polymerisation of allylamine (ppAm) and avoids the need for labelling or chemical modification of the GAGs. The ppAm coats the surface in positively charged amine groups (–NH_2_) [434], thereby allowing for the subsequent immobilisation of negatively charged GAGs. Plasma polymerisation is an established technology involving a vessel containing the vapour of a monomer at low pressure and an energy source (see [435]). Generally, the lower the ratio of power to monomer input rate, the higher the retention of monomer functionality, and in addition substrate temperature and the location of the plasma discharge relative to the substrate have been shown to affect surface chemistry [436]. Heparin immobilised onto ppAm-coated microtiter plates was able to interact with the heparin-binding proteins tumour necrosis factor-stimulated gene-6 (TSG-6), complement factor H, and the chemokine interleukin 8, whereas no functional heparin was present on untreated plates [433]. The positive charge of the allylamine coating, and the fact that the binding of heparin to the surface was salt-strength dependent indicates that the binding is at least partly through an ionic interaction [433,437]. It was also demonstrated that the ppAm coating could be used to create heparin gradients, and as well as heparin, can also support the functional binding of a wide range of GAGs, including CS, HA, and DS, although differences existed in their protein-binding capabilities depending on the surface chemistry to which they were adsorbed [437,438]. Yang et al. [439] have also coated stainless steel with ppAm, in order to develop a heparinised surface with biological function. They found that the heparin-binding surface inhibited the ability of human umbilical artery smooth muscle cells to adhere and proliferate, while enhancing that of human umbilical vein endothelial cells. The heparinised surface was also shown to inhibit thrombosis and promote re-endothelialisation in vivo. Meade et al. [335] translated the cold plasma polymerisation technology onto 3D constructs, developing a novel electrospun scaffold functionalised with GAGs non-covalently attached to the fiber surface via a ppAm coating. Bound GAGs were shown to be biologically active, restoring the ability of EXT1^−/−^ (HS-deficient) mESCs to differentiate down the neural lineage. Use of these scaffolds to investigate how GAGs can control the chondrogenic differentiation of hMSCs would therefore be interesting. 

Whilst the incorporation of GAGs into biomaterials for improved TE strategies is not new, most attempts thus far have relied upon the use of poorly defined, heterogeneous, commercially available, preparations [188,189,190,192,194,335,395,426,428,439,440,441]. Although these strategies have generally proven effective, it is difficult to decipher specific structure-function relationships, and there is the worry that off-target effects may lead to sub-optimal or even detrimental effects [190,442,443,444,445,446]. In addition, the use of natural polymers, generally isolated from animal tissues, is a concern for clinical translation, not only due to the high levels of structural complexity, but also due to potential contamination. As recently as 2008, batches of heparin containing oversulfated CS, caused allergic-type reactions in patients and led to over 100 deaths, highlighting the need for a more controllable method of production [447]; this requirement has also recently been underlined by the identification of significant batch variation in commercially available preparations of HS used in research [448]. In an effort to purify more defined HS preparations, Cool and colleagues have successfully employed a peptide affinity isolation method [163,169,193,195,295]; this technique is suitable for scale-up to meet clinical demand, and has already been shown to be effective for potentiating the bioactivity and bioavailability of a number of growth factors. Further to this, the advancement of GAG modelling, sequencing and synthesis tools over the past decade (see [326,359,360,361]), has also meant that the feasibility of generating synthetic GAG mimetics is progressing rapidly (see [449]). Chemoenzymatic methods using bacterial glycosyltransferases and synthetic UDP monosaccharide donors can now allow for the rapid production of structurally defined HS oligosaccharides [450], where the expression of HS biosynthetic enzymes at high levels in *E. coli* is making the process amenable to scale-up [451]. In addition, a novel strategy, whereby the 6-hydroxyl group of synthetic UDP-GlcNAc or UDP-GlcNS nucleotides is substituted by an azido group, is facilitating the synthesis of GAG mimetics in excellent yields [452]; here the azido moiety is converted to a sulfate group, leading to the production of oligosaccharides with N-sulfates at both the 2- and 6- positions, allowing for close mimicry of natural N-sulfated, 6-O sulfated GlcN residues. Improved understanding of the substrate specificity of C5 epimerase activity has also meant that single product IdoA2S-GlcNS residues can now be irreversibly synthesised (i.e., rather than a mixture of GlcA and IdoA residues) [453]. These improved methods have meant that over 30 synthetic heparin oligosaccharides have now been generated [449]. Ultimately, our ability to uncover structure/function relationships in heparin/HS oligosaccharides is evolving rapidly, and with this, the incorporation of more defined and specific HS sequences into TE strategies, in a cost-effective manner, has now become a realistic goal.

## 7. The Problems Associated with Heparin

The high level of sulfation (and thus negative charge) carried by the GAG heparin, means that it is able to bind, stabilise, and modulate the activity of a wide range of growth factors (see [454]). In addition, the low cost, easy availability, and status of heparin as an already FDA- and EMA-approved product, has meant that researchers are harnessing its properties, preferentially over that of other GAGs (such as HS) to improve growth factor delivery [188,189,192,194,440]. Gaining particular prominence is the use of heparin-loaded delivery vehicles for skeletal TE, where biomaterials decorated with heparin have been extensively studied both in vitro and in vivo, for their ability to reduce the dosing requirements and improve the therapeutic potential of BMPs, such as BMP2 [188,189,194,455,456,457,458,459,460]. Most of these studies have shown very promising results, with heparin-loaded biomaterials improving BMP2 delivery, release, and osteogenic potential. It should be noted, however, that some inhibitory effects of heparin on TGFβ superfamily members (and other proteins) have been reported when high doses of the GAG are used [461,462]. In addition, in some cases the use of heparin-loaded biomaterials have proven ineffective in vivo [192] and the long-term side effects have not been tested. Adding to this, it has been shown in a rat ectopic model, that while collagen sponges soaked with BMP2 and heparin did result in more bone formation compared to sponges soaked with BMP2 alone, the use of an HS variant, rather than heparin, improved bone formation even further [190]. On this basis we hypothesised that while heparin may enhance the retention/activity of BMP2 (or other growth factors) under certain conditions, its binding ‘promiscuity’ means that it may also inhibit other factors that play a role in the repair process, leading to sub-optimal or even deleterious results [266].

**Figure 5:**
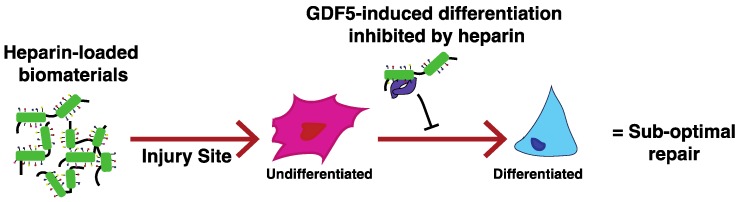
Urging caution over the use of heparin-loaded biomaterials for TE strategies. Heparin has an inhibitory effect on the activity of GDF5. As such, the incorporation of heparin-loaded biomaterials into skeletal TE strategies may have an inhibitory effect on GDF5, which is found naturally at the repair site and that may also be important to the repair process, leading to suboptimal or deleterious outcomes.

In addition to the potential off-target effects from heparin-incorporated biomaterials, the long-term use of heparin as an anticoagulant has also resulted in a number of adverse clinical effects being reported, including thrombocytopenia, vascular reactions and osteoporosis [442,443,444,445]. Cool and colleagues, recently showed that heparin has donor-dependent effects on the stemness and multipotency of hMSCs, and alters a number of signalling pathways associated with hMSC growth and differentiation [446]. This raises a concern regarding the long-term use of heparin in the clinic and its suitability in skeletal (and other) TE strategies. Furthermore, the potential of GDF5 in cartilage TE strategies (see Section 4.1.2.1 above) lead us to investigate the interaction between GDF5 and heparin. We identified GDF5 as a novel heparin/HS-binding protein, and demonstrated that heparin (but not equivalent doses of HS) has a strong and clear inhibitory effect on the biological activity of GDF5, even at doses 10-fold lower than those that would be clinically administered (Figure 4E) [266]; e.g., for patients with venous thromboembolism, the dose of heparin is usually maintained at 0.3 to 0.7 U/mL [463], while 10 nM of heparin used in our study equates to around 0.03 to 0.04 U/mL. In addition, the inhibitory effect of heparin was seen across multiple hMSC donors and in the skeletal cell line ATDC5. Given the importance of GDF5 for skeletal development, our results might help explain the reported increased risk of developing osteoporosis following long-term heparin treatment [442,444], and the variable (and disappointing) results seen with heparin-loaded biomaterials for skeletal repair [190,192]. As illustrated in Figure 5, these data add further caution to the widespread use of unfractionated heparin, both in the clinic and research settings.

Importantly, the HS used in our study did not show the same inhibitory effect as heparin on GDF5 activity (Figure 4E), indicating that HS may be a more suitable, safe and effective alternative for incorporation into TE strategies and stem cell expansion/differentiation protocols. However, it is hard to generalise when HS is so heterogeneous/diverse, and additional studies with better-defined HS preparations will of course be necessary to investigate this further. It should also be noted, that the unfractionated HS preparation we used (from porcine intestinal mucosa) did not significantly promote the activity of GDF5 either. Again, given the sequence diversity of HS [309,328,330], we anticipate that it will be possible to identify HS variants that promote GDF5 activity while retaining other beneficial effects on chondrocyte function/phenotype [266]. Previous work by the Cool/Nurcombe group indicates that isolation of HS variants by their affinity for particular growth factors, enables the selection of saccharides markedly more potent at promoting growth factor activity compared to using unfractionated HS starting material, with its intrinsically high level of compositional heterogeneity [190,193,195,295]. Thus, there is the possibility to potentiate the activity of specific growth factors, to the same extent as heparin, while avoiding the adverse and off-target effects stemming from heparin’s pleiotropic nature. Indeed, our work has revealed that the HS3 variant (selected through its affinity for BMP2 and shown to promote BMP2 activity [193,195]), did not inhibit GDF5 in the same manner as heparin [266]. Therefore, by using this particular HS preparation, there is the possibility of promoting BMP2 activity (as with heparin) without affecting the activity of GDF5, which would also be present at the injury site and be involved in the repair process [259,261,464]. This further highlights the therapeutic utility of developing selective HS variants, rather than using heparin. Interestingly, given that HS3 has a higher level of sulfation compared to unfractionated HS [193], and carries an overall charge more similar to heparin, it might have been expected to also inhibit GDF5 activity. However, this was not the case [266] and suggests that charge density is not the dominant feature in the interaction between GDF5 and heparin/HS, and that GAG structure and sequence may also play a role. 

The clear inhibitory effects of exogenously added heparin on GDF5 activity, also led us to look at whether there was a requirement for endogenous HSPGs for GDF5 binding and localisation [266]. Previous studies have shown that overexpression of *Drosophila* HSPGs (i.e., division abnormally delayed (Dally) and Dally-like protein (Dlp)), result in enhanced *Drosophila* Decapentaplegic (Dpp) (an ortholog of vertebrate BMP2/4) signalling in the wing [465]. However, in HSPG deficient mutants, Dpp signalling is reduced [466]. These results therefore suggest a positive role for HSPGs in BMP signalling, perhaps by acting as a co-receptor for BMPs (as with FGF signalling [467,468]), or by modulating the bioavailability of BMPs at the cell surface. In favour of the latter hypothesis, our data [266] demonstrated that pericellular HSPGs play a key role in localising GDF5 to the cell surface, with a positive correlation between HSPG and GDF5 concentration being observed (Figure 4D). This is in good agreement with results reported for BMP2 [190,469], and further emphasises the importance of HSPGs in regulating the activity of many TGFβ superfamily members. These results then led us to hypothesise that the inhibitory effects of heparin on GDF5-induced activity may be due to the exogenous heparin out-competing the binding of pericellular HSPGs to GDF5; i.e., preventing GDF5 from localising to the cell surface and binding to its receptors to initiate downstream signalling/activity [266]. In agreement with this hypothesis, we were able to show that heparin (but not equivalent doses of HS) inhibited both GDF5 association to the cell surface, and GDF5-induced downstream Smad 1/5/8 signalling. Interestingly, we have recently observed that despite the lack of HS on the surface of HSPG-deficient CHO cells, and the decreased binding of GDF5 to these cells (compared to WT CHO), over a one hour time period, GDF5 signalling appeared similar in both cell lines (B.I. Ayerst, V. Nurcome, A.J. Day, C.L.R. Merry and S. Cool, *unpublished observations*). We therefore suggest that while HSPGs are important for capturing and improving the bioavailability of GDF5 at the cell surface, and perhaps protecting the growth factor from proteolytic degradation, unlike the FGF family of growth factors [467,468], HSPGs are not critical for the interaction between GDF5 and its receptor for downstream signalling.

**Figure 6:**
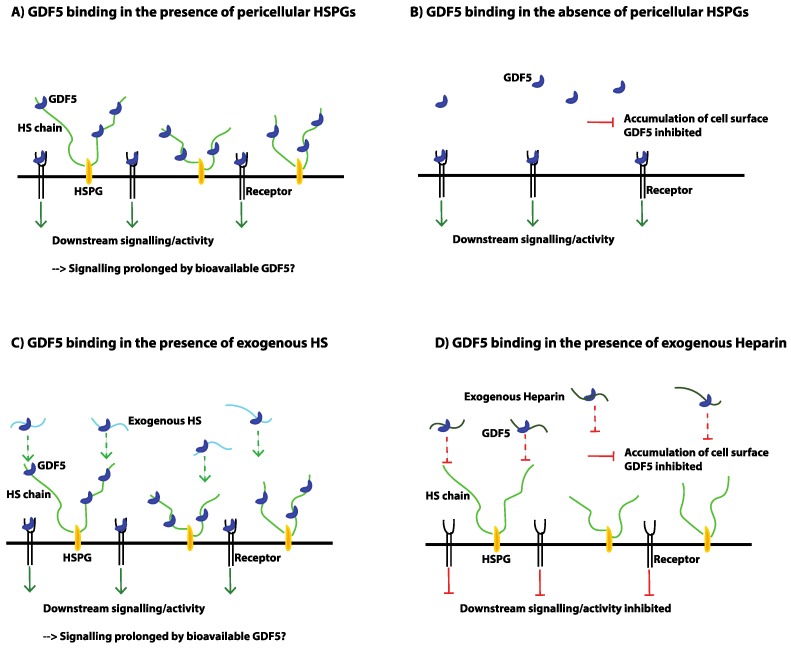
Proposed methods of regulation of GDF5 activity by cell surface HSPGs and exogenously added heparin/HS. GDF5 is captured by HSPGs and accumulates at the cell surface, where it is then available for prolonged receptor binding and activity (**A**). In the absence of HSPGs the accumulation of GDF5 at the cell surface does not occur; GDF5 is still able to bind to its receptors to initiate activity, but the free ligand may be more susceptible to proteolytic degradation, and as a result the duration of downstream signalling and activity may not be as prolonged as in the presence of HSPGs; (**B**). Exogenous HS is able to bind to GDF5, but the higher affinity of cell surface HSPGs and receptors for GDF5 binding, means that the interaction is only transient in nature; cell surface HSPGs and receptors out-compete exogenous HS for the GDF5 interaction and, as a result, similar levels of signalling are seen as in situation part A. However, if a specific high affinity HS is used (at a high enough concentration) then inhibition would be seen (**C**). The high affinity of heparin for GDF5 means that this GAG binds and out-competes cell-surface HSPGs and receptors for binding. Both the accumulation of GDF5 at the cell surface and downstream signalling is inhibited (**D**).

Since the inhibitory effect of exogenous heparin on GDF5 activity cannot simply be explained by a lack of accumulation of GDF5 at the cell surface, we suggest that the high affinity of heparin for GDF5 may also more directly inhibit GDF5-receptor interaction [266]. In addition, heparin has also been shown to inhibit the activity of heparanases [470,471,472]. While treatment with heparanse has been shown to stimulate chondrogenesis in micromass cultures of mouse embryo limb mesenchymal cells, the heparanase antagonist SST0001 (a heparin molecule, modified so that it lacks anticoagulant activity) strongly inhibited chondrogenesis [473]. Heparanase was also found to be over expressed in chondrocytes from patients with hereditary multiple exostoses; a skeletal disorder resulting in the formation of benign cartilagenous tumours. In this sense, the inhibitory effect of heparin on GDF5-induced activity seen in our study may be three fold; firstly the high affinity of heparin for GDF5 may directly inhibit GDF5-receptor interactions; secondly, heparin may bind to GDF5, preventing it from associating with cell surface HS, and therefore limiting its bioavailability; thirdly heparin may inhibit the activity of heparanases, thereby preventing GDF5 from being released from cell surface HSPGs, which is required for its interaction with its receptors and the initiation of downstream signaling (Figure 6). 

The pleiotropic nature and off-target effects of heparin render it, in our opinion, unsuitable for use in TE strategies [442,443,444,445,446]. Importantly, HS-based therapeutics may offer improved outcomes, with a more direct and targeted control of growth factor activity [357]. Indeed, a successful strategy for isolating HS fractions according to their affinity for specific growth factors has been developed [163,193,195,295]. The arrangement of sulfate groups within HS chains are thought to align with basic amino acids within the heparin-binding domain of target proteins [474]. As such, the synthesis of peptides based on heparin-binding domains of proteins, can allow for affinity-based HS purification and the isolation of variants with increased growth factor potency. The technology has already been used to isolate HS variants with increased affinity for BMP2, VEGF_165_, and FGF2, which have all proved more efficacious than unfractionated HS for bone healing, angiogenesis, and stem cell expansion, respectively [163,193,195,295]. 

However, although successful in the cases above, limitations and challenges still exist in the widespread translation of this affinity purification platform. The use of linear heparin-binding peptide sequences is simplistic and not representative of the majority of heparin-protein interactions [158,169,475,476,477,478]. Ultimately, a major obstacle for the use of HS as a therapeutic lies in its vast heterogeneity, including (large) variations in its domain structure, sulfation level/pattern, and chain length [479]. On top of this, simple/effective methodologies for the sequencing of HS (and other GAG) chains are not yet available; thus, making it difficult to determine and interpret structure-function relationships [321]. In addition, the degree of specificity involved in protein-HS interactions is still very much open for discussion [159,328,330,480,481,482], adding doubt over how precisely protein-HS interactions can be controlled in vivo. Another important factor is that binding affinity does not always directly equate to activity, and broad recognition/specificity does not necessarily indicate insignificance [483,484]; further adding to the complexities of developing a standard and effective method for targeted HS therapeutics.

Early on, the discovery of the sequence required for antithrombin III (ATIII) binding indicated that protein-HS interactions may be governed by specific sequences encoded within the primary structure of HS [485]. ATIII requires a 3-O-sulfated pentasaccharide in order to bind HS; i.e., GlcNS/NAc(6S)-GlcA-GlcNS(3S±6S)-IdoA(±2S)-GlcNS(±6S). Removal of the 3-O sulfate reduces the pentasaccharide’s affinity for ATIII by 17,000 fold [486]. However, since this finding, very few specific binding sequences have been identified, perhaps with the exception of FGF2 [155,487], and it has been suggested that the specificity of protein-HS interactions instead exists on many different levels; with some interactions being more specific than others [484]. The importance of sulfation patterning also exists at a chain level as well as at an individual saccharide level, where the distribution of sulfated domains within an HS chain can dictate binding specificity as well as (or in some cases, in preference over) the specific sulfation sequence within those domains [159,488,489]. In support of this, HS chains produced by biosynthetic mutant mice, lacking HS 2-O-sulfotransferase activity, were still able to support FGF2 activity despite lacking 2-O sulfation, apparently because they had compensatory increases in N- and 6-O sulfation that maintained the charge distribution along the HS chain [489]. While HS contains regions of both high and low sulfation, known as NS and NA domains, respectively, heparin can be seen as a hypersulfated version of HS, with virtually continuous sulfation along its chains, i.e., forming essentially one long NS domain [479,490]. The overall high level of sulfation in heparin may therefore mask selectivity, leading to its pleiotropic nature. It has been suggested that in the case of some heparin/HS-protein interactions little specificity exists, and that instead overall charge density is the predominant factor in determining binding [483,491]. 

It is therefore likely that the extent of HS-binding specificity varies among different proteins, and that overall charge density, sulfate patterning, and sequence specificity on HS chains can all play an important role. In addition, while electrostatic interactions between sulfate/carboxyl groups in the GAG chains and surface exposed positively charged arginine/lysine residues in the protein are undoubtedly important [492], evidence also suggests that non-ionic interactions, such as van der Waals forces, hydrogen bonding, and aromatic ring stacking, can also play a role [328,481,492,493,494,495,496]. Interestingly, a recent study has suggested that the presence of residues such as asparagine or glutamine can help to identify heparin/HS binding sites [496]; where these uncharged residues confer specificity on the interaction. If correct, this could revolutionise the identification of HS-protein binding sites, and thus facilitate the design of targeted HS-based therapeutics. 

While experimental and modelling techniques are rapidly advancing, we are still only in the infancy of being able to fully characterise protein-GAG interactions. The high level of heterogeneity, conformational flexibility, and uncertainty over binding mechanisms has meant that it is hard to model and identify interactions in a high-throughput and effective manner. In addition, it appears that the level of specificity involved in protein-GAG interactions is not universal, and high affinity HS variants may not always lead to the desired outcome. As such, the development of targeted HS therapeutics may be a more complex task than first envisioned. However, the fact that a degree of specificity does seem to exist for certain proteins, means that the use of HS variants, rather than heparin, to more tightly control protein activity, is still an exciting and realistic prospect. The development of more advanced modelling and sequencing tools, along with the further refinement of biomaterials for the delivery and application of HS variants, will however first be required, before the full potential of HS glycotherapeutics can be realised.

## 8. Conclusions

The low cost, easy availability, high level of sulfation, and status of heparin as an already FDA- and EMA- approved product, has meant that it has largely been the GAG of choice for TE applications. While the use of heparin has generally proven effective, the long-term side effects of its incorporation into biomaterials has not been tested, and in some cases the use of heparin-loaded biomaterials have proven ineffective in vivo. While heparin may enhance the retention/activity of certain growth factors under certain conditions, its binding ‘promiscuity’ means that it may also inhibit other factors that play an important role in the repair process, leading to suboptimal or even deleterious effects. These concerns have been highlighted by our recent work, indicating that exogenous heparin has a strong inhibitory effect on the activity of growth differentiation factor 5 (GDF5), a growth factor which plays a critical role in cartilage/skeletal formation and homeostasis. This is particularly worrying, given the increasing incorporation of heparin into skeletal TE strategies. Overall, there is growing evidence cautioning the use of heparin both in the clinic and in TE applications, and emphasising the need to transition to using more specific GAGs (e.g., HS derivatives or synthetics), with better-defined structures and fewer off-target effects, if optimal therapy is to be achieved. Importantly, the advancement of GAG modelling, sequencing and synthesis tools over the past decade are starting to allow for this transition; enabling us to move away from the use of heterogenous undefined GAG preparations, and opening up more advanced opportunities for the use of GAGs in a more controlled and defined manner. 

## Figures and Tables

**Table 1:** Growth factors implemented in chondrogenesis and associated PGs/GAGs.

Molecule Family	Molecule	Proposed Function during Chondrogenesis	Reference	Associated PGs/GAGs	Reference
**FGF** ^1^	FGF2	Enhances proliferation and chondrogenic potential during expansion.	[59,148,149,150,151]	Role of HSPGs in FGF-receptor binding has been extensively studied; HSPGs play an important role in FGF-receptor signalling by facilitating ligand-receptor oligomerisation.	[155,156,157,158,159]
		Negative effect on matrix deposition and differentiation.	[151,152,153]		
		Addition during expansion primes cells for hypertrophy.	[149,151]		
		Prolongs lifespan of MSCs.	[154]		
	FGF9	Increases matrix production early on, but then promotes matrix resorption and hypertrophy.	[152]	CS sulfation patterns have also been implicated in articular cartilage formation and expression has been co-localised with FGF2. Perlecan can only deliver FGF2 to its receptors after its CS chains have been removed.	[160,161,162]
		However, also reported to promote matrix production and delay terminal hypertrophy.	[151]		
	FGF18	Suppresses proliferation and promotes matrix production.	[151,164]	Exogenous HS can be used to improve hMSC expansion.	[163]
		Delays terminal hypertrophy.	[151]		
**TGF**β	TGFβ1/3	Promotes chondrogenic differentiation of MSCs. Considered a main chondrogenic inducer of MSCs, however, leads to chondrocyte hypertrophy.	[36,37,38,39,41,75,128,165]	TGFβ1 but not TGFβ3 has been shown to bind to HS; effects of the interaction remain conflicting.	[166,167,168,169]
		TGFβ3 better supports chondrogenic differentiation than TGFβ1.		TGFβ binds to the small leucine rich PGs, decorin, biglycan and fibromodulin, but via their protein core; CS/DS chains interfere with this binding.	[170]
**BMP**	GDF5	Important role in joint formation and organisation of articular cartilage; GDF5 expressed in healthy pre-hypertrophic cartilage, but not as OA develops; GDF5 dominant negative mutation results in articular cartilage degeneration.	[23,171,172,173,174,175]	Heparin binding sequence predicted.	[182]
		Increases cartilaginous ECM production in vitro.	[176,177,178,179]		
		Supplementation with TGFβ3 shown to promote hypertrophy. However, a combinatorial study with TGFβ1, BMP2 and GDF5 suggest that it is the TGFβ actually promoting hypertrophy.	[180,181]		
	BMP2/4/6/7	Promotes chondrogenic differentiation of MSCs, especially when used in combination with TGFβ. BMP2/7 indicated as particularly useful for inducing chondrogenesis. However, most studies indicate that BMP supplementation also leads to hypertrophy.	[153,181,183,184,185,186]	Use of heparin/HS to potentiate the activity of BMPs has been widely studied; especially in the case of BMP2 for bone TE.	[187,188,189,190,191,192,193,194,195]
**Wnt**	Wnt3a, Wnt5a	Promotes chondrogenic differentiation.	[196,197,198]	Glypican3 is strongly linked to the Wnt pathway; HS chains bind to Wnts with different affinities to fine-tune access to Wnt receptors; 6-O-desulfation of HS reduces ability of Glypican1 HS chains to bind Wnt, and therefore facilitates Wnt-receptor interaction.	[200,201,202]
		Inhibits hypertrophy.	[199]		
		However, Wnt5a also reported to promote hypertrophy during early stages of differentiation.			
	Wnt11	Promotes chondrogenic differentiation and hypertrophy.	[203]		
	Wnt4, Wnt8	Inhibits chondrogenic differentiation. Promotes hypertrophy.	[196,204]		
	Wnt9a	Inhibits chondrogenic differentiation. Inhibits hypertrophy.	[205]		
**IGF**	IGF1	When used in combination with TGFβ3 collagen I production is reduced.	[183]	Heparin/HS/DS stimulate the release of free and bioactive IGF1 from IGF binding proteins	[207]
		Promotes hypertrophic differentiation.	[206]		
**PTHrP**	PTHrP (1–34) isoform	Inhibits TGFβ induced hypertrophic differentiation.	[39,153,208,209]	PTHrP is activated by Ihh signalling (feedback loop). HS binds Ihh and negatively regulates signalling.	[210,211]

**^1^** FGF, fibroblast growth factor; HSPG, heparan sulfate proteoglycan; CS, chondroitin sulfate; DS, dermatan sulfate; TGFβ, transforming growth factor beta; BMP, bone morphogenetic protein; GDF, growth differentiation factor; Wnt, Wingless-type MMTV integration site; IGF, insulin like growth factor; PTHrP, parathyroid hormone-related peptide.

**Table 2:** Components of the TGFβ Smad-dependent canonical signalling pathway.

Molecular Category	TGFβ Sub-Family Pathway ^1^	BMP Sub-Family Pathway
Ligands	TGFβs, ActivinsGDF8/9/10/11, BMP3, Nodal	BMP2/4/5/6/7/8/9/10, GDF1/3/5/6/7, MIS
Type II receptors	TβRII, ActRIIA, ActRIIB	BMPRII, ActRIIA, ActRIIB
Type I receptors	ALK4, TβRI (ALK5), ALK7	ALK1/2, BMPR1A (ALK3), BMPR1B (ALK6)
R-Smad	Smad2/3	Smad1/5/8
Co-Smad	Smad4	Smad4
I-Smad	Smad7	Smad6/7

**^1^** Alternative protein names are listed in brackets. TβR, TGFβ receptor; MIS, muellerian inhibiting substance; BMPR, BMP receptor; ActR, activin receptor; ALK, activin receptor-like kinase. Table adapted from [226].
